# Turing Instability-Driven Biofabrication of Branching Tissue Structures: A Dynamic Simulation and Analysis Based on the Reaction–Diffusion Mechanism †

**DOI:** 10.3390/mi9030109

**Published:** 2018-03-02

**Authors:** Xiaolu Zhu, Hao Yang

**Affiliations:** 1 College of Mechanical & Electrical Engineering, Hohai University, Changzhou 213022, Jiangsu, China; zhuxiaolu@hhu.edu.cn; 2 Changzhou Key Laboratory of Digital Manufacture Technology, Hohai University, Changzhou 213022, Jiangsu, China; 3 Jiangsu Key Laboratory of Special Robot Technology, Hohai University, Changzhou 213022, Jiangsu, China

**Keywords:** reaction–diffusion mechanism, 4D biofabrication, 3D morphogenesis, self-organization of cells

## Abstract

Four-dimensional (4D) biofabrication techniques aim to dynamically produce and control three-dimensional (3D) biological structures that would transform their shapes or functionalities with time, when a stimulus is imposed or cell post-printing self-assembly occurs. The evolution of 3D branching patterns via self-assembly of cells is critical for the 4D biofabrication of artificial organs or tissues with branched geometry. However, it is still unclear how the formation and evolution of these branching patterns are biologically encoded. Here, we study the biofabrication of lung branching structures utilizing a simulation model based on Turing instability that raises a dynamic reaction–diffusion (RD) process of the biomolecules and cells. The simulation model incorporates partial differential equations of four variables, describing the tempo-spatial distribution of the variables in 3D over time. The simulation results present the formation and evolution process of 3D branching patterns over time and also interpret both the behaviors of side-branching and tip-splitting as the stalk grows and the fabrication style under an external concentration gradient of morphogen, through 3D visualization. This provides a theoretical framework for rationally guiding the 4D biofabrication of lung airway grafts via cellular self-organization, which would potentially reduce the complexity of future experimental research and number of trials.

## 1. Introduction

Three-dimensional (3D) printing in health science mainly aims to mimic biological functions [1,2,3]. However, there are difficulties in controlling the shape and functions of the printed 3D bio-constructs/materials because of the reorganization of the printed bio-construct via cellular migration and proliferation [3,4,5]. Thus, the regulation of the transformation or functionalities of 3D biological structures over time is critical for achieving the 3D bio-construct with the expected shape and functions [5,6]. To reach this goal, a four-dimensional (4D) biofabrication technique might be utilized, which aims to create dynamic 3D biological structures that can transform their shapes or functionalities with time when an external stimulus is imposed or when cell post-printing self-assembly occurs [5,7]. 4D biofabrication can be used for the bioprinting of vascularized tissues with high resolution of spatial and temporal control [6], shape-memory polymer (SMP)-based scaffolds for bone tissue engineering [8], hollow self-folding tubes based on printing of cell-laden shape-morphing hydrogels [9], and fabrication of 3D cellular structures within tubes, self-folded by thermosensitive shape-changing polymer films [10]. The evolution of 3D patterns with a specific geometry via self-assembly of cells, is critical for 4D biofabrication of artificial organs or tissues with branched geometry.

Recent research on organ morphogenesis has described an elaborate pattern of branching phenomena [11,12]. In the lung, two primary forms of branching have been identified: side-branching and tip-splitting. The branching occurs in sequence: first, side-branching creates the primary stalks, then there is a change of mode to tip-splitting. The kidney developmental program encodes the intricate branching and organization of approximately 1 million functional units (nephrons) [13]. These phenomena have been hypothesized to be under genetic control [11,14,15]. How genes could possibly act to produce these patterns is still not clear. However, the coding of branch patterning is possibly simplified by the repeated use of a branching mechanism, as in Mandelbrot’s fractal model and other elegant algorithms [16,17,18,19,20]. 

The reaction–diffusion (RD) system is one of the basic systems which can describe the evolution of biological systems [21,22]. In 1952, Alan Turing described morphogens, patterns, and different forms, about biological systems [21] and proposed the Turing principle of spatial patterns, showing that diffusion could lead to instability. This type of instability can be called Turing instability. The RD model based on Turing instability has shown its potential power in simulating the dynamic process of molecules and tissue morphogenesis [23,24,25] with time. In our previous study of the dynamics of 3D biopattern formation [2,3,26,27], we showed that Turing instability drove the evolution of the self-organization of 3D multicellular patterns. Turing instability can induce spatial patterns in mathematical models, such as stripes, spots, hole patterns, and more complicated 3D patterns, and is applied to model biological patterning phenomena in natural animals and plants [28,29] or the tissue morphogenesis [2,3,4]. The rational control of the RD system leads to a flexible guiding methodology for artificial 3D tissue morphogenesis. There have been several typical mathematical models that study lung branching phenomena. Menshykau et al. [30] proposed a Schnakenberg-type Turing model based on the molecular interactions between fbroblast growth factor 10 (FGF10), Sonic hedgehog (SHH), and Patched (Ptc), which reproduces the experimentally observed wild-type branching pattern. Celliere et al. [31] then added FGF9 to the model, to simultaneously predict the emergence of smooth muscles in the clefts between growing lung buds, and vascular endothelial growth factor A (VEGFA) in the distal sub-epithelial mesenchyme. However, their model is not a model of morphogenetic growth, but mainly a model of periodic spots appearing surrounding the lung bud. Further, a ligand–receptor-based Turing model was used to predict the embryonic areas of outgrowth and it supports branch outgrowth [25], implicating only two interacting factors (the ligand FGF10 and its receptor fibroblast growth factor receptor 2b (FGFR2b)). Nevertheless, FGF10 is expressed only in the mesenchyme, while FGFR2b is expressed only in the epithelium, so there was a particular geometry restricted for variables in this model that may lose the underlying process in complex biological system. Other types of modalities mainly involve the mechanics-based model [32], which outlines the pattern formation from a purely mechanical viewpoint. For example, cytoskeletal tension mediated by Rho signaling plays a role during cleft formation in lung branching. Another form of the mechanics-based model is the 3D vertex model [33], which enables the quantitative simulation of multicellular morphogenesis based on single cell mechanics involving various cellular activities, such as cell contraction, growth, rearrangement, division, and death. Yet, these mechanics-based models may neglect some key underlying molecular processes in the biochemical environment. 

Generally, the rational prediction-based RD mathematical model is flexible and is the most commonly used methodology, which is also a suitable method to guide the 3D or 4D biofabrication by predicting the dynamic evolution of 3D self-organized microstructures over time. The RD model commonly requires a morphogen pair [34] (an activator–inhibitor pair) in which the inhibitor diffuses more rapidly than its activator. In recent studies of lung branching morphogenesis based on the RD model [35,36], a four-variable partial differential equation (PDE) according to Meinhardt [17] was utilized to describe the reaction and diffusion of morphogens involved in creating branched lung development. Usually, bone morphogenetic protein-4 (BMP4) can serve as an activator, matrix gamma-carboxyglutamic acid protein (MGP) can serve as an inhibitor, and the substrate *S* can be fibroblast growth factor 10 (FGF10). In the microenvironment during lung development, FGF10 is usually present, so the variable *S* describing this type of growth factor has to be incorporated into the basic model. In order to describe the 3D structure of the branches assembled by the differentiated cells, we need a variable representing the local concentration of differentiated cells. Here, *Y* is the variable that indicates the concentrations of cell differentiation markers (*Y* = 0 indicates no differentiated cell while *Y* = *Ya* (*Ya* is a threshold value and typically 0 < *Ya* ≤ 1) indicates totally differentiated cells). Therefore, these four parameters, respectively, have their own different roles in describing these RD multicellular systems and none could be omitted. We have previously presented the model and simulation results on the emergence of side-branching and tip bifurcation [37]. In this paper, we attempt to comprehensively and systematically study the features of the model and simulate the time-dependent phenomena of stalk growing, side-branching, and tip bifurcationg by using a 3D branching evolution model based on RD dynamics. In addition to the simulation and discussion on the evolution of 3D structures, we also present and discuss the law governing the change of 3D bifurcation patterns in 2D parameter domains, the fabrication style under an external concentration gradient of morphogen, and the limitations of this prediction model. These studies are more extensive when compared to our previous paper [37]. The simulated branching morphogenesis, both in longitudinal (growth) and in transverse directions of the stalk, is demonstrated and analyzed. The simulation results and analysis provide a theoretical framework of the 3D and 4D fabrication of branching structures for lung or kidney in a cellular self-organization manner.

## 2. Mathematical Model

The mathematical model based on Turing’s reaction–diffusion (RD) dynamics, according to Meinhardt [17], involves four concentration variables; activator, inhibitor, substrate, and cell differentiation marker. Here, the four variables are labeled as activator (*A*), inhibitor (*H*), substrate (*S*) and cell differentiation marker (*Y*). The mathematical expressions are as follows:

(1) $$\frac{\partial A}{\partial t}=\frac{cA^{2}S}{H}-\mu A+D_{A}\nabla^{2}A+\rho_{A}Y$$

(2) $$\frac{\partial H}{\partial t}=cA^{2}S-\nu H+D_{H}\nabla^{2}H+\rho_{H}Y$$

(3) $$\frac{\partial S}{\partial t}=c_{0}-\gamma S-\varepsilon YS+D_{S}\nabla^{2}S$$

(4) $$\frac{\partial Y}{\partial t}=dA-eY+\frac{Y^{2}}{1+fY^{2}}$$

The model postulates four spatially continuous variables, each of which is a spatio-temporal function. *A*, *H*, *S*, and *Y* are four concentration variables indicating the concentrations of activator, inhibitor, substrate, and cell differentiation marker (*Y* = 0 indicates no differentiated cell, while *Y* = *Ya* (*Ya* is a threshold value and typically 0 < *Ya* ≤ 1) indicates totally differentiated cells [28]). In the model, *μ*, *v*, *γ*, and *e* represent the first-order degradation rate of *A*, *H*, *S*, and *Y* respectively; *D_A_*, *D_H_*, and *D_S_* are the diffusion coefficient of activator, inhibitor, and substrate; the differentiated *Y* cells secrete activator *A* and inhibitor *H* at the rates of *ρ_A_* and *ρ_H_* respectively; *c* is the positive rate of activator *A* in the autocatalytic reaction; *c*_0_ and *ε* are the production and consumption rates of substrate *S*. *cA*^2^*S/H* describes the autocatalytic generation of *A* under the action of *S* and *H*; *cA*^2^*S* describes the generation of *H* during the interaction of *A* and *S*; *dA* describes cell differentiation markers generated by activator *A*; *D_A_*▽^2^*A*, *D_H_*▽^2^*H*, and *D_S_*▽^2^*S* describe the diffusion of activator, inhibitor, and substrate, respectively. Equations (1)–(4) are also denoted as *A*-equation, *H*-equation, *S*-equation, and *Y*-equation, respectively, in this study. High concentrations of activator *A* produces cell commitment *Y* (the *dA* term in the *Y*-equation, e.g., Equation (4)). When the concentration of activator *A* exceeds a certain critical value, cell differentiation (for example, *Y* = *Ya* indicates that cells have been differentiated) is irreversibly activated. The reason of this could be explained by three steps. Step 1: The *A* will increase because of self-production until it reaches a certain value, causing ∂Y/∂t > 0 because of the term *dA* in *Y*-equation, and then *Y* will increase with time because of ∂Y/∂t > 0. Step 2: The ∂Y/∂t cannot always be larger than zero because the term $-eY+Y^{2}/\left(1+fY^{2}\right)$ will start to less than zero when *Y* increases to a certain large value (here *e* = 0.1, *f* = 10) while the term *dA* has a bounded value due to the *A* inhibited by *H* cannot unrestricted increase (here *d* = 0.008). Thus, $\partial Y/\partial t=dA-eY+Y^{2}/\left(1+fY^{2}\right)$ will tend to be increasingly smaller with continually larger *Y*. Step 3: ∂Y/∂t will continue to decrease and reach zero when the *Y* reaches a certain value, e.g., ∂Y/∂t = 0. From this time, *Y* reaches the steady-state value and will not change with time because of ∂Y/∂t= 0. Here, all physical quantities are dimensionless. In this study, the activator could be BMP4, the inhibitor could be MGP, and the substrate is FGF10, according to the literature [36,38]. The diffusion rate of the BMP4, is lower than that of the inhibitor MGP because the molecular weight of BMP4 is bigger than that of MGP, which is in accordance with the parameter setting (*D_A_* < *D_H_*) in the model.

At the beginning of the simulation, almost all positions in the volume were set to *Y* = 0, meaning that the positions did not contain a differentiated cell, but a small region within the center of the whole computing domain is set to *Y* = 1, representing the initial lung stalk bud. Then, growth happened from the initial sites converting from *Y* = 0 to *Y* = *Ya*, in the presence of high concentrations of activator (the +*dA* term in the *Y*-equation). The tissue was represented by positions at which *Y* = *Ya*. The mathematical model was solved by the finite difference method in the MATLAB platform (MathWorks, Inc., Natick, MA, USA), and the parameters were traversed in a certain range, which was also implemented for solving other RD models [26,27,39]. Three-dimensional data were displayed by a 3D visual software (Voxler, trail version). The simulation ran on a computer with a 16-core CPU and at least 32 Gigabytes of memory and a 2 Terabyte hard disk. Usually, one set of parameters needed from about several hours to ten hours to complete.

All of the simulations in this study are highly reproducible. The simulated 3D branching structures will remain the same through multiple simulations as long as the set of parameter values does not change.

## 3. The Evolution of Side Branching

Lung development begins with side branches emerging in rows around the circumference of the parent/primary branch. The parent branch elongates, and new side branches bud off. In this model, the primary branch (*Y*-stalk) grows through the positive feedback of the peaks of activator *A*. Also, inhibitor *H* is produced proportionally to the activator distribution, and *H* diffuses from a high-concentration region, to a low-concentration region. 

In this model, the activator peak forms because of the positive feedback of *A* on itself. The activator peak then produces differentiated cells *Y* (via the +*dA* term in *Y*-equation). The region occupied by activated *Y* indicates the geometry of the *Y*-stalk. The initial micro-region, represented by the isosurface of the *Y* value, is usually not able to be a standard spherical geometry because the *A* and *H* concentrations have a 5% fluctuation, and thus the propagate of *Y* = *Ya* from the initial point to surrounding region is not spherical in symmetry. So, the initial region consisting of differentiated cells (*Y* = *Ya*) will be geometrically polarized, which leads to the formation of a slightly elongated *Y*-stalk. Y cells consume *S* (the −*εYS* term in *S*-equation), while the region without *Y* cells consumes less *S*. Thus, the gradient of *S* forms, which is the main driver of activator migration, so the newly formed activator peak will move in the direction of the gradient of *S* concentration. On the other hand, the inhibitor *H* is produced in response to the activator peak and diffuses (the terms–*cA^2^S* and $D_{H}\nabla^{2}H$ in *H*-equation). The stalk can elongate because both ends of the stalk have peaks of activator *A* that produce new *Y* cells, and *H* diffuses to the side of the primary branch serving as lateral inhibition, resulting in a filamentary elongation of the *Y*-stalk rather than a circular or isotropic expansion.

**Figure 1:**
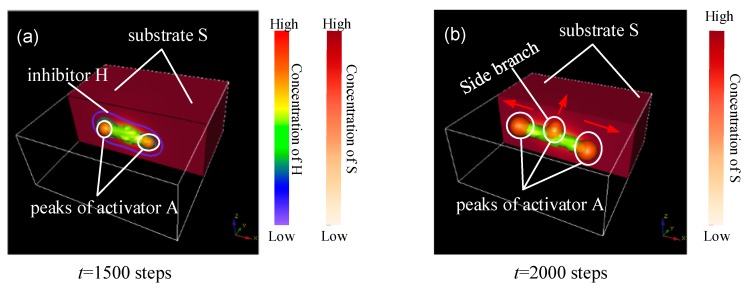
Concentration distribution of *A*, *H*, and *S* in side branching. (**a**) Simulation result at *t* = 1500 steps; (**b**) Simulation result at *t* = 2000 steps. The green part is the spatial distribution of the cellular differentiation marker *Y*; the orange spherical structure is the peaks of activator *A*; the contour lines indicated the distribution of *H*; the red rectangle is the spatial distribution of the substrate *S*. Substrates are actually distributed in the area formed by the white rectangular border. To clearly show the intrinsic association of *A*, *H*, and *S* concentration profiles, half of the substrate was hidden. The numbers under the images denote the step number in the simulation. Parameters: *c* = 0.04, *μ* = 0.3, *v* = 0.03, *ρA* = 0.03, *ρH* = 0.0001, *c*_0_ = 0.02, *γ* = 0.02, *ε* = 0.042, *d* = 0.008, *e* = 0.1, *f* = 10, *D_A_* = 0.1, *D_H_* = 0.26, *D_S_* = 0.06.

The peaks of activator *A* appear at both ends and at the middle of the parent branch, are attracted by the substrate (the term $+cA^{2}S/H$ in *A*-equation), and migrate to the direction of high *S* concentration (arrows in Figure 1a,b). When the concentration of activator *A* exceeds a certain critical value, cell differentiation is irreversibly activated (the detailed reason is elucidated in Section 2). The spatial distribution of the cellular differentiation marker *Y* is indicated by the green part in Figure 1. The activator peaks with high concentration produce cell commitment *Y* because of the term *dA* in Equation (4), the activator peaks migrate in the direction of the arrows, and a side branch forms in the middle position of the *Y*-stalk, as shown in Figure 1b. Therefore, the *Y*-branches appear in the region where high concentrations of activation peaks migrate. In addition, it is clear that *S* concentration is lower at the location of the *Y*-stalk, but is relatively higher away from the *Y*-stalk (the −*εYS* term in *S*-equation). 

**Figure 2:**
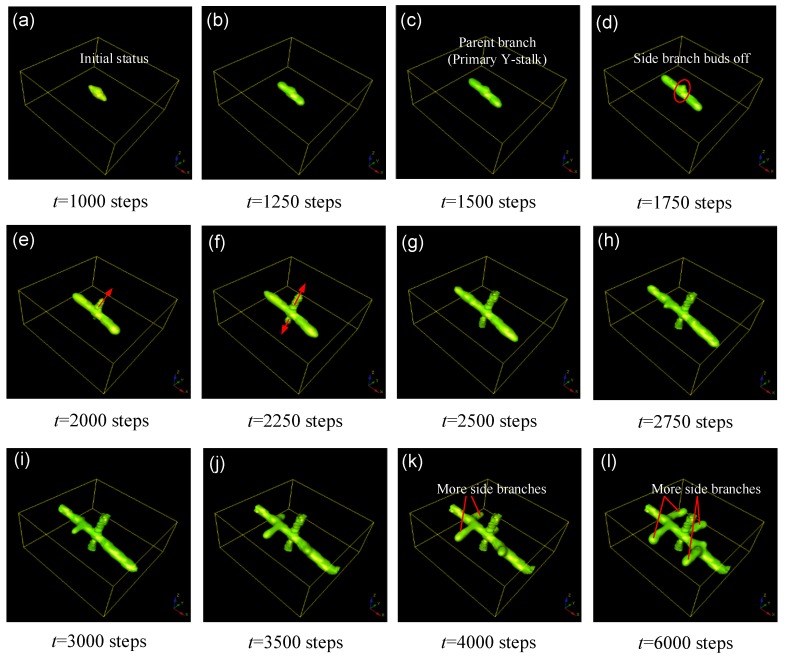
Simulation results for side-branching behaviors in 3D. The presented structure is the spatial distribution of the cellular differentiation marker *Y*. The numbers under the images denote the step number in the simulation. Parameters: *c* = 0.04, *μ* = 0.38, *v* = 0.04, *ρA* = 0.03, *ρH* = 0.0001, *c*_0_ = 0.02, *γ* = 0.03, *ε* = 0.042, *d* = 0.008, *e* = 0.1, *f* = 10, *D_A_* = 0.1, *D_H_* = 0.26, *D_S_* = 0.06.

The *Y*-stalk elongates because the *H* that diffuses to the side of the primary branch serves as lateral inhibition that is presented by the contour lines in Figure 1a, which results in vimineous elongation of the *Y* stalk, as shown in Figure 1. The elongation of the activator peak continues to generate differentiated cells *Y*. Thus, the elongated primary branch is formed (Figure 2). However, *Y* cells consume *S* (refer to the −*εYS* term in the *S*-equation), and thus *S* concentration becomes lower at the location of the *Y*-stalk but remains relatively higher away from the *Y*-stalk. Therefore, a gradient of *S* forms, which is the main driver of activator migration, because the activator *A* is always seeking the substrate *S* according to the term $+cA^{2}S/H$ in the *A*-equation. Consequently, the newly formed activator peak will migrate in the direction from low *S* concentration to high *S* concentration, as indicated by the arrows in Figure 2e,f. Along the direction perpendicular to the primary stalk, side branches form and grow longer when the attraction of the substrate overcomes the inhibition (Figure 2f–h). Next, more branches appear along the direction of the primary *Y*-stalk elongation (Figure 2h–l). Each activator peak in 3D on the *Y*-stalk leads to a side branch, as that activator peak migrates perpendicularly into regions of high concentrations of *S*, far away from the main *Y*-stalk where *Y* cells have depleted *S* (refer to the −*εYS* term in the *S*-equation).

## 4. Tip Bifurcation

**Figure 3:**
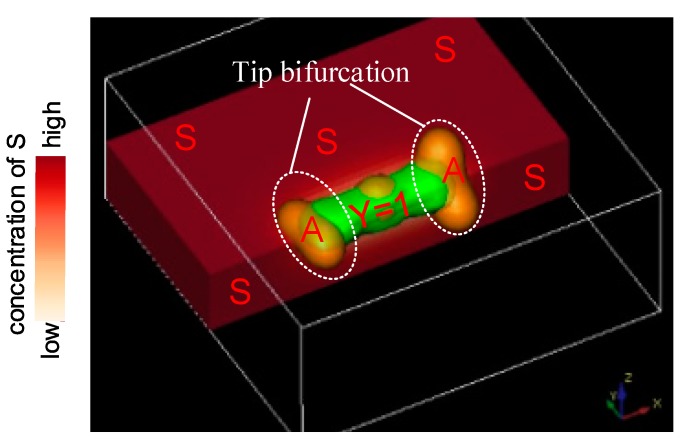
Schematic illustration of the profile of activator concentration along the direction transverse to stalk elongation. The tip bifurcation direction deviates from the primary stalk extension direction.

In the simulation, tip bifurcation is another typical branching type in addition to the side-branching. Tip bifurcations occur at the tips of a stalk, not in middle positions as during side-branching. Tip bifurcation begins with the lateral extending of the activator *A* concentration profile. Since substrate *S* in the current position is exhausted, the activator no longer moves in the former direction and has to look for new substrate *S* in new directions deviating from the former stalk extension direction (Figure 3).

There are two lateral extending behaviors of activator *A* concentration profiles in the horizontal and vertical planes, respectively (Figure 3). The white border represents the boundary of the simulation domain; the red rectangle represents substrate *S*, which distributes throughout the simulation domain space. Here, only about 1/4 is taken for description, and the deeper the color, the higher the substrate *S* concentration. The green part represents the differentiated cells (*Y* = *Ya*, here *Ya* = 0.5). The orange ellipsoid indicates the isosurface of activator *A*. It can be seen from Figure 3 that the concentration of substrate *S* is lower around the differentiated cells (the term −*εYS* in the *S*-equation leads to the local *S* decreases), whereas activator *A* extends to the high *S* direction (the term *cA*^2^*S/H* in the *A*-equation leads to a large value of *A* in the region of high *S*). A high concentration of substrate *S* exists in the domain perpendicular to the original direction, which results in the transversal expanding of activator *A*. The concentration of activator *A* therefore exhibits a flat, long profile.

**Figure 4:**
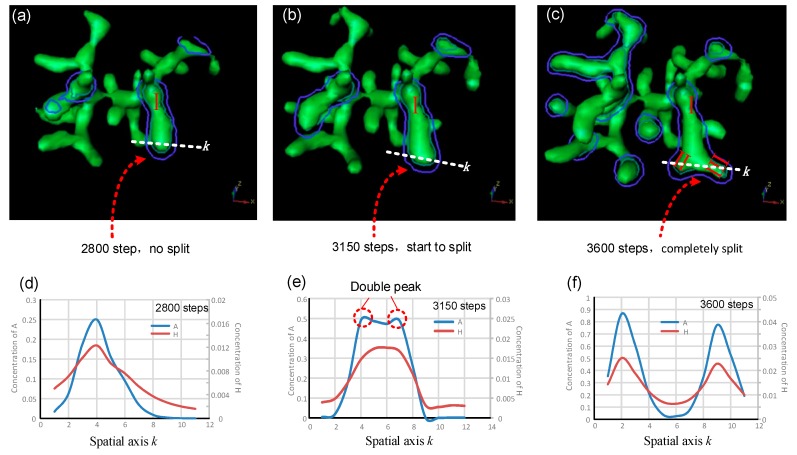
Simulation results for branching behaviors in typical tip bifurcation. (**a**–**c**) Spatial distributions of the cellular differentiation marker *Y* after 2800, 3150, and 3600 steps, respectively. Variation tendencies of the activator and inhibitor along the spatial axis *k* after 2800, 3150, and 3600 steps, respectively (**d**–**f**). The tip bifurcation could be examined along the spatial axis *k* on the plane defined by the blue, closed contours. The coordinates on the horizontal and vertical axes are dimensionless. Parameters: *c* = 0.04, *μ* = 0.8, *v* = 0.08, *ρ_A_* = 0.03, *ρ_H_* = 0.0001, *c*_0_ = 0.02, *γ* = 0.02, *ε* = 0.042, *d* = 0.008, *e* = 0.1, *f* = 10, *D_A_* = 0.1, *D_H_* = 0.26, *D_S_* = 0.06.

In order to study the distribution and relative time-dependent changes of activator *A* and inhibitor *H* in the transverse direction perpendicular to that of stalk extension, typical bifurcation (tip-splitting) was simulated. Here, we selected the simulation results after 2800, 3150, and 3600 steps respectively, and drew the corresponding 3D graphics of multicellular morphologies for analysis. The specific numerical values and variation tendencies of activator *A* and inhibitor *H* along a certain spatial coordinate axis *k* are shown in Figure 4.

Figure 4a–c shows 3D representations of the cell differentiation marker *Y* isosurface. Stalk I extends longitudinally in the original direction (Figure 4a). The tip of stalk I then becomes large and flat, indicating that the tip is expanding perpendicularly, transverse to the original direction (Figure 4b). Finally, the tip of stalk I completely splits, and stalk I is divided into two daughter branches, branch II and branch III (Figure 4c). The symmetrical center plane of stalk I serves as a tangent plane (planes defined by blue and green contours, Figure 4a–c). A line crossing the largest width through the tip of stalk I is made on the tangent plane (white dotted lines, Figure 4a–c) and it serves as the spatial axis *k* in Figure 4d–f. Figure 4d–f show the concentration distributions and change laws of activator *A* and inhibitor *H* along the spatial axis *k*. Since inhibitor *H* is generated by activator *A*, the concentration of inhibitor *H* is relatively high in the large *A* region. *A* and *H* have roughly the same change laws along the spatial axis *k* when stalk I extends in the longitudinal direction (Figure 4d). When the tip of stalk I starts expanding transversely (Figure 4b), the concentration distribution profile of activator *A* appears as two peaks (Figure 4e). Meanwhile, inhibitor *H* maintains a unimodal distribution pattern at 2800 and 3150 steps and only later appears as two peaks (Figure 4d–f). Consequently, the concentration distribution change of inhibitor *H* lags behind that of activator *A*. The delayed inhibitor peak resembles a knife, significantly reducing the concentration of the activator in the corresponding regions and forcing the activator to divide into two peaks with lateral expansion. After the tip splits completely, the inhibitor peak also divides into two peaks, and its distribution along the spatial axis *k* is almost the same as that of activator *A* (Figure 4c,f). Further research for the 4D biofabrication may rely on the 3D culture techniques [3,27,40] that are essentially constructing and regulating the dynamic structures composed of numerous cells. 

## 5. The Evolution of Tip Bifurcation

**Figure 5:**
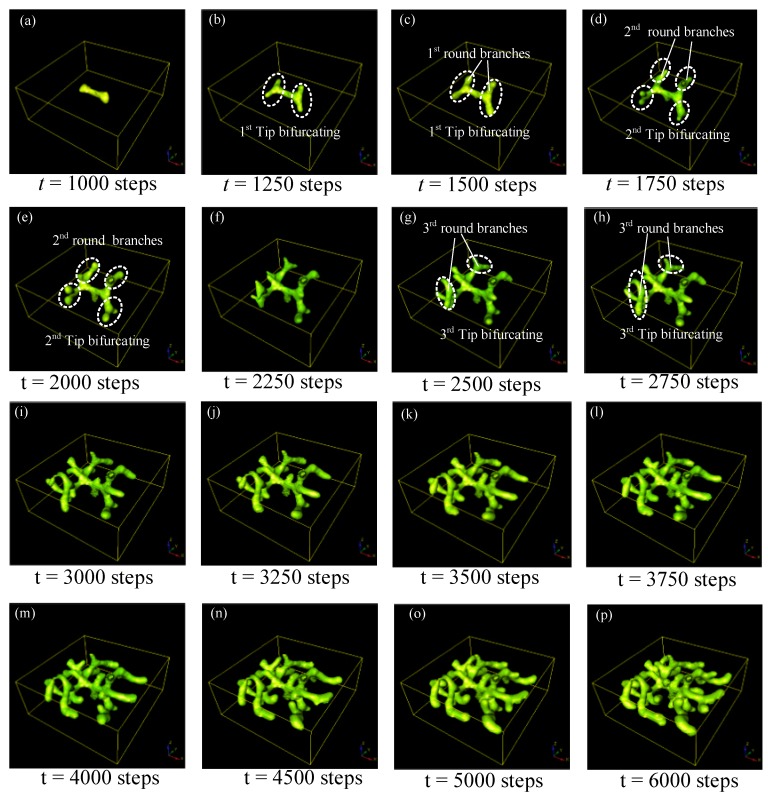
Simulation results for tip-splitting behaviors in 3D. The presented structure is the spatial distribution of the cellular differentiation marker *Y*. The numbers under the images denote the step numbers in the simulation. Parameters: *c* = 0.04, *μ* = 0.38, *v* = 0.04, *ρ_A_* = 0.03, *ρ_H_* = 0.0001, *c*_0_ = 0.02, *γ* = 0.03, *ε* = 0.042, *d* = 0.008, *e* = 0.1, *f* = 10, *D_A_* = 0.1, *D_H_* = 0.26, *D_S_* = 0.06.

The whole evolution process of tip bifurcation was investigated by simulation based on the following parameter values: *c* = 0.04, *μ* = 0.48, *v* = 0.06, *ρ_A_* = 0.03, *ρ_H_* = 0.0001, *c*_0_ = 0.02, *γ* = 0.02, *ε* = 0.042, *d* = 0.008, *e* = 0.1, *f* = 10, *D_A_* = 0.1, *D_H_* = 0.26, *D_S_* = 0.06. With the depletion of substrate *S*, only a small amount of substrate *S* remains in the leading segment of stalk growth, and the activation peak would exhaust substrate *S* in this region. To look for new substrate *S*, the activator peak naturally expands transversely after a period of time, resulting in tip bifurcation events (Figure 5a–c). After the first-round tip bifurcation, the daughter branches generated could be called the 1st round branches (Figure 5c). With the evolution of this RD process, the second round tip bifurcation occurs at the outer tips of the first round branches (Figure 5d,e). As time progresses, another round of bifurcation occurs at the tips of branches, which generates the thirrd round branches (Figure 5g–h). Following that, more and more sub-branches are generated via the tip splitting process, and these sub-branches grow longer and generate further sub-branches (Figure 5i–p). This creates a hierarchical 3D structure with several-level geometric features. The number of branches and sub-branches increases very rapidly, especially in the later period of the evolution, because tip splitting behaviors of all sub-branches happen simultaneously. This could be a parallel fabrication manner for creating branched 3D structures.

**Figure 6:**
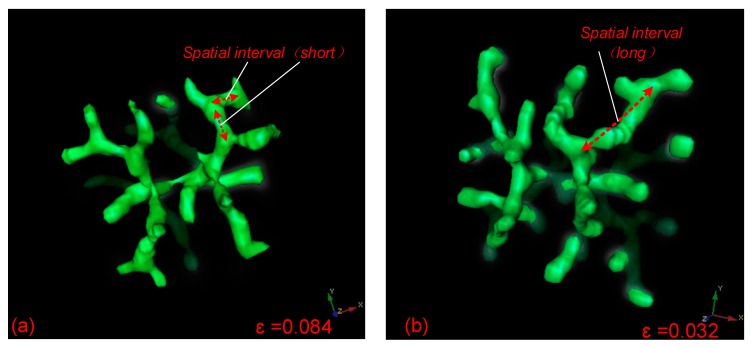
Comparison of the segment lengths of spatial intervals between sequential branching events with different parameter values. (**a**) When ε = 0.084, the spatial interval between sequential branching events is relatively short; (**b**) when *ε* = 0.032, the spatial interval between branches is relatively long. Parameters: *c* = 0.04, *μ* = 0.8, *v* = 0.08, *ρ_A_* = 0.03, *ρ_H_* = 0.0001, *c*_0_ = 0.02, *γ* = 0.02, *d* = 0.008, *e* = 0.1, *f* = 10, *D_A_* = 0.1, *D_H_* = 0.26, *D_S_* = 0.06, *ε* = 0.084 or 0.032.

For the tip bifurcation events illustrated in the simulation, the distance between a bifurcation point and the next adjacent bifurcation point is defined as the spatial separation of bifurcation events in this study. It is determined by the distance of the extension of the leading activator peak before the next bifurcation event. In order to develop the Turing instability-driven 4D biofabrication method, we needed to investigate the size control approach for these 3D structures. Controlling the distance of spatial separation in 3D branching structure could be achieved by adjusting the consumption (or depletion) rates *ε* of Substrate *S*. When the *ε* is large, only a small amount of *S* remains in the leading segment of the stalk growth, and the activation peak will promptly exhaust *S* in this region when continuously moving forward. To look for new substrate *S*, the activator peak will naturally expand transversely after a very short period of time, resulting in bifurcation events with small spatial separation (Figure 6a). In contrast, when *ε* is smaller, the activation peak propagates transversely and produces branches at a lower rate. Considering the low consumption rate of substrate *S*, there is a large amount of substrate *S* residues in front of the growing stalk. Therefore, the activator peak will continue to expand along the original direction rather than expanding transversely until the *S* at the current position is depleted, resulting in large spatial separation of bifurcation events (Figure 6b). A comparison of bifurcation events with short and long spatial separation based on different parameters is shown in Figure 6. It can be seen that the spatial interval of the bifurcation events is relatively short when *ε* = 0.084, and the bifurcation events have a relatively longer spatial interval when *ε* is reduced to 0.032.

## 6. Distribution Law of Bifurcation Patterns in Parameter Domains

The activator and the inhibitor are usually biomolecules such as proteins. In this study, the activator could be BMP4, the inhibitor could be MGP, and the substrate could be FGF10, according to the literature [36,38]. In 3D simulations, branch patterns and spatial separation between branch points can be altered by changing one or more parameter values. The degradation rates of protein molecules (BMP4/MGP/FGF10) are relatively easy to control in the experiment by adding a corresponding amount of chemical substances such as modulator, enzyme inhibitor, or antagonist for these proteins [41,42,43,44]. The diffusion rates of the proteins (BMP4/MGP/FGF10) can be changed by varying the density of the 3D hydrogel matrix in the experiments. The degradation rates are also influenced by the extracellular matrix, i.e., the hydrogel, and one form of “degradation” expected for both BMP-4 and MGP could probably be the sequestration into the extracellular matrix [45]. Therefore, these parameters in this model can be connected to corresponding conditions in the experiments, and the first-order degradation rates of *A*, *H*, *S*, and *Y* could be candidates for control parameters. 

**Figure 7:**
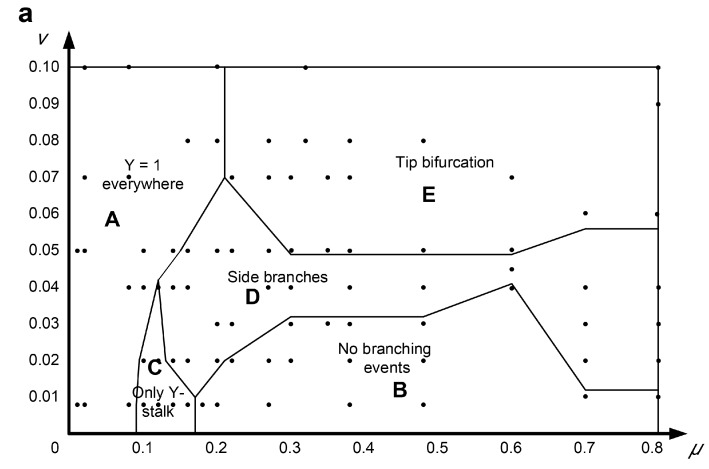

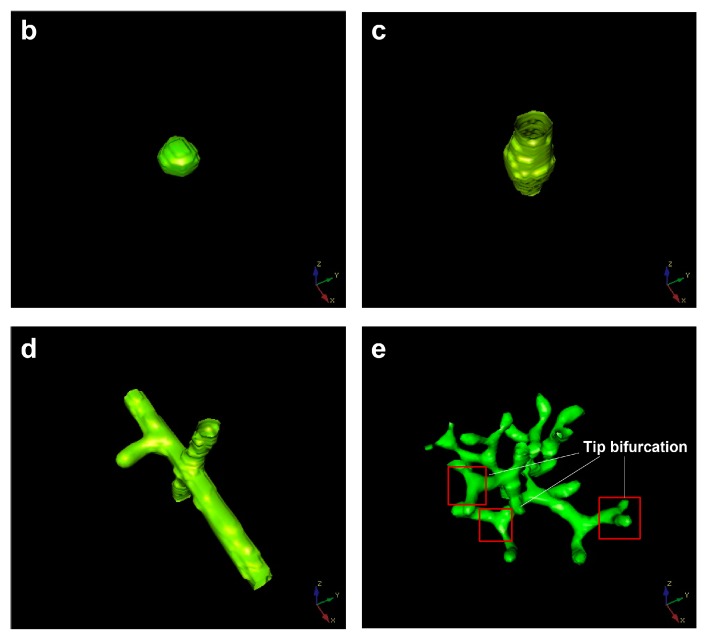
(**a**) The distribution laws of the types of branching behaviors within the *μ*-*v* parameter domain according to the simulation results. The *μ*-*v* parameter domain is composed of five different branch patterns, which are: *Y* = 1 everywhere pattern (spatial spillover type, area A), no branching events (no branch type, area B) pattern, only branches (only parent branch type, *Y* represents the *Y* variable in the equation, area C) pattern, side branches (side branches type, area D) pattern, and tip bifurcation (tip splitting type, area E) pattern. The multicellular morphologies corresponding to areas B–E are shown in graphs (**b**–**e**) respectively.

Here, *μ* and *v* were investigated within the ranges of *μ* ∈ [0, 0.8], *v* ∈ [0, 0.1], in which variant 3D patterns emerged, as shown in Figure 7. The parameter *μ* represents the abscissa and the parameter *v* represents the *y*-axis; *μ-v* are combined to form the 2D parameter domain. A series of points in the simulation domains were calculated to explore the states of branch patterns by altering the parameters *μ* and *v* individually or by simultaneously changing both together. This systematic study of branch pattern conversions under a parameter domain provides a potentially reasonable adjustment method for the reconstruction of branches with different organizational structures.

The *μ-v* parameter domain is composed of five different branch patterns, which are: *Y* = 1 branches present everywhere (spatial spillover type, Figure 7, area A), no branching events (no branch type, Figure 7, area B), only branches (only parent branch type, *Y* represents the *Y* variable in the equation, Figure 7, area C), side branches (side branches type, Figure 7, area D), and tip bifurcation (tip splitting type, Figure 7, area E). The multicellular morphology corresponding to each pattern is also shown in Figure 7. Area A, located on the left of the parameter domain, represents the *Y* = 1 everywhere pattern. In this region, the corresponding patterns are very thick and almost fill in the entire simulation domain. Additionally, increasing μ can switch the *Y* = 1 “everywhere” pattern to any other pattern, except for the “no branching” pattern. Area B is in the lower-right of the *μ-v* domain, indicating that the “no branching events” pattern exists for a smaller range of parameter *v* (Figure 7, area B). When *μ* ∈ [0.2, 0.6], the boundary curve of area B gradually increases, and the *v* of the highest point is about 0.04. When *μ* ∈ [0.6, 0.7], the boundary curve of area B decreases sharply and finally becomes horizontal. The “no branching events” pattern resembles a sphere (Figure 7b) and is essentially an isotropic or circular expansion of the stalk, rather than the normal filamentary elongation. This is because the inhibitor *H* diffuses to the circumference of the stalk and results in circular inhibition instead of lateral inhibition. Area C, representing only the “*Y*-stalk” pattern as shown in Figure 7c, occupies the smallest area of the five patterns in the *μ*-*v* domain. The pattern easily switches to other modes following slight changes in *μ* or *v*. Area D, the “side branches” pattern, is above area C. The border of area D gradually widens to 0.45 in the case of *μ* > 0.6. The reasons for pattern formation in area D are detailed in Section 3. The most significant feature of the “side branches” pattern is that area D is adjacent to all four other regions, indicating that the “side branches” pattern can easily change to any other mode by changing the parameters *μ* and *v*. This provides rich and flexible guiding information for a future experimental study. Area E represents the “tip-splitting” pattern and is an approximately rectangular area located in the upper part of the *μ*-*v* domain. It occupies roughly 1/3 of the entire parameter domain. When *μ* ≥ 0.02 and *v* ≥ 0.05, the “tip bifurcation” pattern completely encompasses the *μ*-*v* domain. Area E is adjacent to areas A and D, indicating that the “tip bifurcation” mode can switch to the *Y* = 1 “everywhere” pattern via a reduction in *μ*, or it can transform into the “side branches” pattern via a reduction in *v*. It could be an effective guidance for the practical manipulation of the degradation rates (*μ* and *v*) of protein molecules.

Based on the simulation results of the *μ-v* domain, we found that the greatest effect of parameter *μ* was on the thickness of the branches, and that parameter *v* was positively correlated with the number of branches. A large *μ* indicates a high degradation rate of activator *A*, resulting in a smaller branch width and a lower value of the initially formed activator singlet. The initially formed activator singlet migrates toward new substrate *S*, and the *Y* cells form branches on the path passed by the activation peak. Therefore, the formed branch is narrow in the case of a narrow activator singlet. The activation peak must extend transversely to a sufficient width to accommodate two new simultaneous activation peaks before activation peak splitting. Here, tip bifurcation can be produced by broadening the activation peak region or narrowing the inhibitor domain of the activator peak. A large *v* leads to rapid degradation of inhibitor *H*, which produces a smaller inhibitor domain of the activator peak. Therefore, the activation peak is more likely to split, increasing the number of formed branches. 

## 7. The 3D Morphological Change under an External Concentration Gradient of Activator

**Figure 8:**
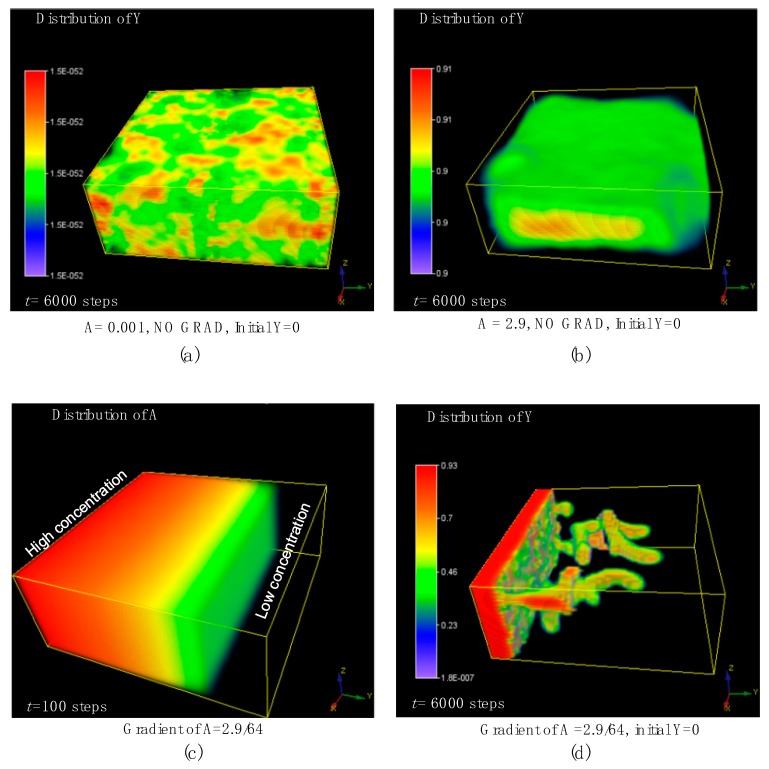
The effect of the external imposed concentration gradient of activator *A*. (**a**,**b**) show the simulation results of the control group without any concentration gradient by setting the average *A* equals to 0.001 (**a**) and 2.9 (**b**), respectively. (**c**,**d**) show the simulation results of the external concentration gradient of *A* at 100 steps (**c**) and the branching structure under this concentration gradient of *A* (**d**). The simulation was performed in a 64 × 64 × 32 domain. Initially, all positions in the whole volume were set to *Y* = 0, meaning that all the positions did not contain any differentiated cells. Parameters: *c* = 0.04, *μ* = 0.48, *v* = 0.06, *ρ_A_* = 0.03, *ρ_H_* = 0.0001, *c*_0_ = 0.02, *γ* = 0.02, *ε* = 0.042, *d* = 0.008, *e* = 0.1, *f* = 10, *D_A_* = 0.1, *D_H_* = 0.26, *D_S_* = 0.06.

At the beginning of this simulation, all positions in the whole volume were set to *Y* = 0, meaning that all the positions did not contain any differentiated cells, which is different from the above simulation settings in this study. Here, we first ran the simulations without any concentration gradient of morphogens by setting the initial average *A* to equal a typical low value of 0.001, and a typical high value of 2.9. As a result, there were no branching patterns that emerged in the 3D domain as shown in Figure 8a,b. In contrast, once an external concentration gradient of *A* was imposed at the initial time, the simulation results were totally changed, and the stable tip-bifurcation type of 3D branching patterns emerged. According to the simulation results, we found that the concentration gradient of activator generated 3D tip-splitting branch structures with no need of the initially placed differentiated cells in the center of the domain. This could be a useful discovery because it would greatly simplify future experiments, with no need to prepare and place the differentiated cells just in the small center region within the 3D matrix domain at the initial time. This generated branching pattern, under the external concentration gradient, remains robust even if the initial setting of *A*, *H*, *S* are slightly changed after many simulations.

## 8. Selected Morphogens and Their Roles in Lung Development

This model postulates four variables: activator *A*, inhibitor *H*, substrate *S*, and commitment *Y*. These variables should correspond to real biophysical conditions. The activator carries out autocatalysis and induces commitment (*Y* = *Ya*). The inhibitor inhibits the production of activator and avoids an explosion of the autocatalytic activator. Both activator and inhibitor require the substrate for their production, and the substrate may come from other cell types nearby [46]. The potential candidates for the morphogens can be proposed by using the functional definitions of each morphogen, as stated above.

FGF10 is expressed in the mesenchyme of the lung, directing the directional growth or migration of individual buds, and promotes lung endoderm proliferation and migration in vitro [11,47,48]. In our model, the substrate *S* is expressed in the mesenchyme that is geometrically complementary to the *Y*-stalks. *Y* cells consume *S* (the −*εYS* term in *S*-equation), while the region without *Y* cells consumes less *S*, which is consistent with a previous study [25]. Thus, the gradient of *S* forms and is the main driver of the activator migration. The newly formed activator peak will move in the direction of the gradient of *S* concentration. The moving peaks of activator *A* produce new *Y* cells, and the *H* diffusing to the side of *Y*-stalk serves as lateral inhibition. Consequently, the *Y*-stalk is further elongated. This is exactly the role attributed to FGF10 [47,48]. 

BMP4 has several features that qualify it as a potential activator morphogen in this model. It is expressed in the terminal epithelial buds and rises at the tips of new branches [47,49,50]. BMP4 has an auto-stimulatory positive feedback in lung development [51], and this feature is consistent with the positive feedback on *A* production in the *A*-equation of this mathematical model. In in vitro organ culture, exogenous BMP4 enhanced epithelial cell proliferation and significantly increased the number of terminal branches [52]. This feature is consistent with the dynamics that the activator promotes the commitment of cells in this mathematical model. 

MGP is a well-known inhibitor of BMPs [38,53,54]. MGP is an antagonist of BMP4 that is highly expressed in lungs and is regulated by activin receptor-like kinase 1 (ALK1) [55]. BMP4 also induces the expression of MGP [38]. Therefore, the relation between BMP4 and MGP is consistent with the interaction between activator and inhibitor described in our model. BMP4 expression was markedly upregulated in the epithelium by FGF10 [47,56], which is in accordance with substance *S* inducing the expression of activator (refer to the term $+cA^{2}S/H$ in the *A*-equation), as described in this model. Therefore, BMP4 and MGP were able to act as an activator–inhibitor pair, with FGF10 as the substrate.

## 9. The Limitations of this Model

The model in this study has several limitations: The model describes a highly stylized situation for how the morphogen pair processes, interacts with a substrate chemical and cell differentiation marker, and can produce a 3D structure similar to that of lung branching. However, the simulation model still presents differences with respect to the physiological development of the lung. For example, this model has been simplified to have only one type of cell, i.e., the epithelium, while complex reciprocal interactions between epithelial, endothelial, and interstitial cells are involved in physiological lung formation in vivo [57]. A further limitation of this model is that it deals strictly with reacting and diffusing (RD) chemical morphogens, ignoring the critical role of mechanical factors in lung development [57,58,59]. The absence of mechanical forces probably makes this model incomplete and unable to embrace all the effects present in the physiological environment. However, it has been suggested that the mechanically induced morphogenesis and chemotactic effect-induced morphogenesis can be regarded abstractly as the mechanisms of local activation and lateral inhibition (LALI) [60], which would make these effects also amenable to this model.

Although this model can predict the outward appearance of 3D branching patterns, it does not incorporate mechanisms that could lead to the formation of hollow tubes. The biological literature suggests that the mechanisms behind tubulogenesis may depend on fluid pressure and fluid–mechanical interactions. Lubarsky & Krasnow [61] claimed that liquid secretion is an essential step in tube formation and expansion. So, our current biochemical model will ultimately have to be extended to include mechanical factors, although the mechanical factors may partially act through biochemical morphogens. The variable *Y* is a biomarker that could correspond to proteins on the cell surface. In this model, we did not consider the diffusion and chemotactic effect of this biomarker which may result in cellular migration, but the ratio of diffusion to chemotactic migrations of cells/biomarkers could be an important factor leading to the formation of hollow cavities in the branches [27,38].

## 10. Summary

Based on the Turing RD mechanism, a 3D simulation of the mathematical model describing bifurcation phenomena in biology, including concentrations of activator, inhibitor, substrate, and cell differentiation marker were studied. This 3D simulation model can predict the evolution of side-branching and tip bifurcation of a 3D multicellular structure and also could facilitate the biofabrication of 3D branching structures under a tailored concentration gradient of a single morphogen. In this Turing instability-driven 4D biofabrication method, the control of the geometric parameters of these 3D structures could be achieved by adjusting the consumption (or depletion) rates ε of the substrate *S*. This study lays a potential foundation for guiding the biofabrication of branched structures of lung airways via 3D cellular self-organization from the perspective RD framework, which is expected to reduce the complexity of future experimental research and number of trials.
